# Effects of balance taping using kinesiology tape in a patient with moderate hallux valgus: A case report

**DOI:** 10.1097/MD.0000000000005357

**Published:** 2016-11-18

**Authors:** Sun-Min Lee, Jung-Hoon Lee

**Affiliations:** a Department of Occupational Therapy, College of Rehabilitation Science, Daegu University, Gyeongsan-si; b Department of Physical Therapy, College of Nursing and Healthcare Sciences, Dong-Eui University, Busan, Republic of Korea.

**Keywords:** balance taping, elastic therapeutic tape, hallux valgus angle

## Abstract

**Background:**

Hallux valgus, an increased angle of lateral deviation in the big toe, can cause pain and difficulties in balancing and walking. This study aimed to investigate the effects of balance taping using elastic therapeutic tape on moderate hallux valgus.

**Methods:**

When she walked with shoes, she complained of pain over the medial eminence of the hallux metatarsophalangeal (MTP) joint. Balance taping using kinesiology tape was applied for 3 months (average, 16hours/d) to both big toes of a 26-year-old woman with moderate hallux valgus.

**Results:**

On the right side, the hallux valgus angle (HVA) decreased from 21° to 14° and the intermetatarsal angle (IMA) decreased from 15° to 14.5°. On the left side, the HVA decreased from 22° to 11° and the IMA decreased from 15° to 12°. Furthermore, the patient was able to walk long distances in shoes without pain in the medial eminence of the hallux metatarsophalangeal joint.

**Conclusion:**

This study suggested that repeated balance taping with kinesiology tape could be used as a complementary treatment method for moderate hallux valgus.

## 1 Introduction

Hallux valgus is defined as an abnormal increase in the lateral deviation angle of the metatarsophalangeal (MTP) joint of the big toe toward the second toe.^[1]^ The severity of hallux valgus can be determined using radiological angles, specifically, the hallux valgus angle (HVA: the angle between the lines bisecting the first metatarsal bone and the first proximal phalanx of the hallux in the anteroposterior direction) and the intermetatarsal angle (IMA: the angle between the 1st and 2nd metatarsal shafts). The HVA is defined as follows: normal ≤15°, mild <20°, moderate 20° to 40°, and severe ≥40°.^[2]^ The IMA is defined as: normal <9°, mild <14°, moderate 14° to 20°, and severe >20°.^[3]^ Hallux valgus can change foot kinematics^[4]^ and causes difficulties in balance and walking.^[5]^ This case report presents the effects of repeated application of balance taping using kinesiology tape in a female patient with moderate hallux valgus.

## 2 Case report

**Figure 1:**
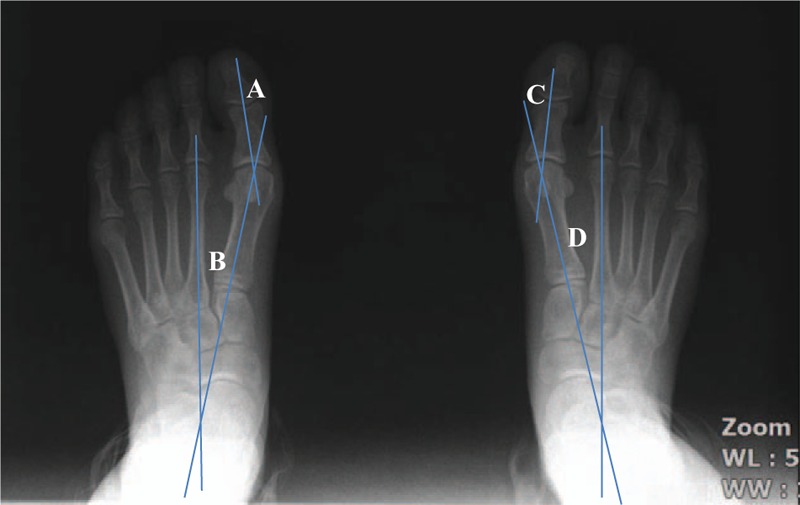
Initial weight bearing radiographic view of both feet (A, left hallux valgus angle; B, left intermetatarsal angle; C, right hallux valgus angle; D, right intermetatarsal angle).

A 26-year-old woman had bilateral moderate hallux valgus. When she walked with shoes, she complained of pain over the medial eminence of the hallux MTP joint, but she had not received any specific treatment. In the initial assessment of radiographic angles, the HVA and IMA on the right side were 21° and 15°, respectively. The HVA and IMA on the left side were 22° and 14°, respectively (Fig. 1). Written informed consent was obtained from the patient for publication of the case report and the use of associated images.

**Figure 2:**
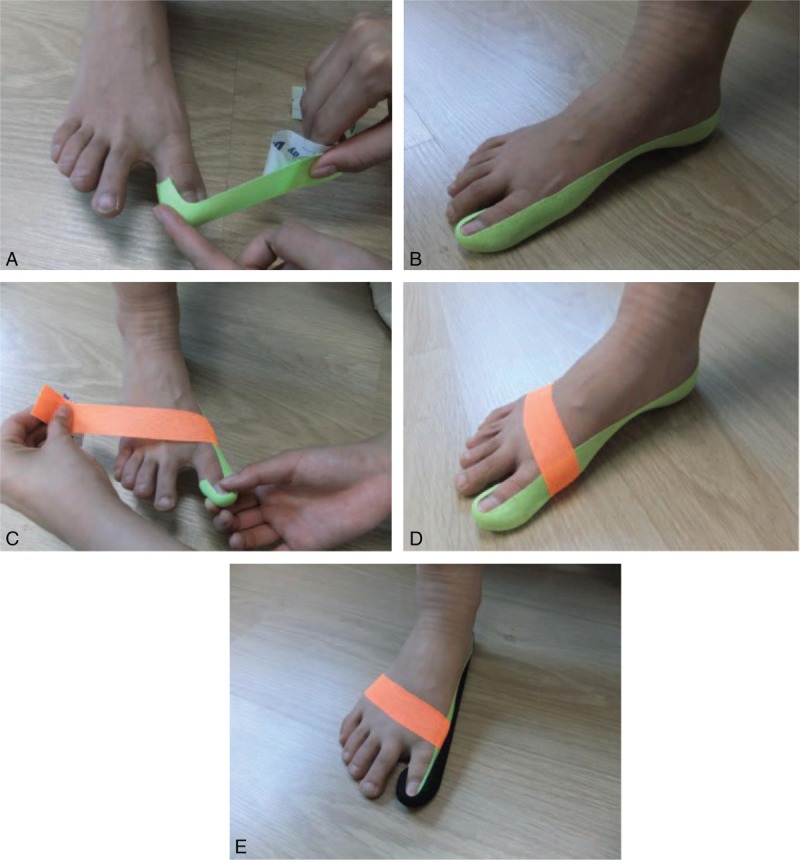
Balance taping using kinesiology tape for right hallux valgus (A and B, applied from the medial aspect of the big toe to the heel; C and D, applied from the base of the hallux metatarsophalangeal joint across the dorsum of foot to the lateral side of the 5th metatarsal bone; E, applied again from the medial aspect of the big toe to the heel).

To reduce hallux valgus, balance taping using kinesiology tape was applied for 3 months (average, 16 hours/d). As the first phase of balance taping for hallux valgus, I-shaped elastic tape of 2.5 cm width (BB TAPE, WETAPE Inc, Paju, Korea) was applied from the medial aspect of the big toe to the heel with a stretch of approximately 30 to 40%, while manually abducting the big toe (Fig. 2A and B). In the second phase, while the big toe was manually abducted to provide mechanical effects that encourage the base of the great toe to turn laterally (toward the second toe), I-shaped elastic tape of 2.5 cm width was applied from the base of the hallux MTP joint across the dorsum of foot to the lateral side of the 5th metatarsal bone, with a stretch of approximately 30% to 40% (Fig. 2C and D). In the third phase, to reinforce mechanical correction in the abduction of the great toe, the same method as the first stage was applied again (Fig. 2E).

To prevent skin irritation, I-shaped elastic tape was applied without any stretch at the ends of the tape (approximately 2–3 cm).^[6]^ The balance taping was removed within 24 hours and reapplied every day, regardless of the presence or absence of skin itching.^[6,7]^ As a result, there was no skin irritation or other adverse event.

**Figure 3:**
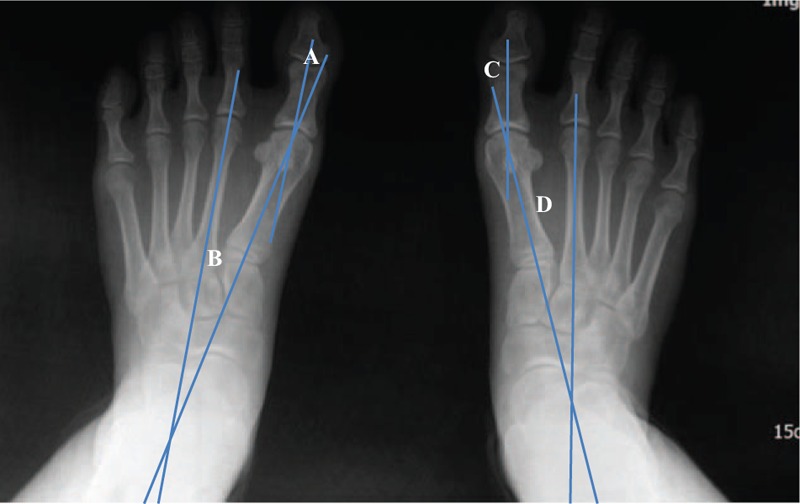
Final weight bearing radiographic view of both feet (A, left hallux valgus angle; B, left intermetatarsal angle; C, right hallux valgus angle; D, right intermetatarsal angle).

After applying balance taping for 3 months, the following changes in radiological angles were observed: on the right side, HVA decreased from 21° to 14° and IMA decreased from 15° to 14.5°; on the left side, HVA decreased from 22° to 11° and IMA decreased from 15° to 12° (Fig. 3). Following treatment, the patient did not feel pain on the medial eminence of hallux MTP joint, even when walking for a prolonged time in shoes.

## 3 Discussion

This case study demonstrates that the repeated application of balance taping for 3 months reduced hallux valgus. Repeated application of balance taping in 3 stages is thought to have had a mechanical correction effect on hallux valgus: in the first and third stages, the laterally deviated big toe was held in abduction; in the second stage, the base of the big toe was turned laterally.

Because the kinesiology tape was applied with a stretch of approximately 30% to 40% in order to produce the mechanical effect of hallux abduction, it is thought that the decreased elasticity of the tape after balance taping resisted the deviation of the hallux toward the second toe. Even if the hallux were to deviate toward the second toe, the tension used during application produces a recoil effect of the kinesiology tape,^[8]^ which may have acted to abduct the hallux and gradually reduce the hallux valgus. However, while the HVA recovered to a normal angle, the IMA did not. Further research into the methodological aspects of balance taping is required.

The application of elastic therapeutic tape used in this study is known to have a mechanical corrective effect on malalignment of the pelvis^[9]^ and shoulder,^[8]^ in addition to preventing injuries^[10]^ and alleviating pain^[6,7,11,12]^ and increasing lymphatic circulation.^[13]^ Although there were differences with the present study in the method and duration of tape application, Jeon et al^[14]^ reported that applying kinesiology tape 15 times in 4 weeks for patients with moderate hallux valgus effectively reduced the HVA from 21.95° to 18.74°.

Although the causal relationship between pain and alignment is unclear, alignment is one of several factors that can cause mechanical pain.^[15]^ Hence, restoring alignment by reducing hallux valgus with repeated balance taping is thought to have helped eliminate pain in the medial eminence of hallux MTP joint, which the patient had previously experienced when walking while wearing shoes.

Based on the results of this case study, repeated balance taping using kinesiology tape may be used as a complementary treatment method for moderate hallux valgus. Further research needs to be conducted to determine whether the balance taping will be effective for patients with more severe symptoms, along with comparative studies with other conservative therapies.
